# CircORC2 is involved in the pathogenesis of slow transit constipation via modulating the signalling of miR‐19a and neurotensin/motilin

**DOI:** 10.1111/jcmm.16211

**Published:** 2021-02-24

**Authors:** Yuan‐Yuan Wang, Rui‐Yun Lu, Ji Shi, Shuai Zhao, Xia Jiang, Xiaosong Gu

**Affiliations:** ^1^ Academy of Medical Engineering and Translational Medicine Tianjin University Tianjin China; ^2^ Department of General Surgery Hebei Key Laboratory of Colorectal Cancer Precision Diagnosis and Treatment The First Hospital of Hebei Medical University Shijiazhuang China

**Keywords:** bowel movement, circRNA‐ORC2, miR‐19a, motilin, neurotensin, slow transit constipation

## Abstract

In this study, we aimed to investigate the role of circORC2 in modulating miR‐19a and its downstream signalling during the pathogenesis of STC. In this study, three groups of patients, that is healthy control (HC) group, normal transit constipation (NTC) group (N = 42) and slow transit constipation (STC) group, were, respectively, recruited. RT‐PCR and Western blot analysis were exploited to investigate the changes in the expression levels of miR‐19a and circORC2 in these patients, so as to establish a circORC2/miR‐19a signalling pathway. The basic information of the patients showed no significant differences among different patient groups. Compared with the HC group, concentrations of neurotensin (NST) and motilin (MLN) were both significantly reduced in the NTC and STC groups, especially in the STC group. Also, miR‐19a level was highest, whereas circORC2 level was lowest in the STC group. Furthermore, circORC2 was validated to sponge the expression of miR‐19a, and the transfection of circORC2 reduced the expression of miR‐19a. Meanwhile, MLN and NST mRNAs were both targeted by miR‐19a, and the transfection of circORC2 dramatically up‐regulated the expression of MLN and NST. On the contrary, the transfection of circORC2 siRNA into SMCs and VSMCs exhibited the opposite effect of circORC2. Collectively, the results of this study established a regulatory relationship among circORC2, miR‐19a and neurotensin/motilin, which indicated that the overexpression of circORC2 could up‐regulate the levels of neurotensin and motilin, thus exerting a beneficial effect during the treatment of STC.

**Abbreviations**
HC: healthy control
MLN: motilin
NST: neurotensin
NTC: normal transit constipation
SMCs: smooth muscle cells
STC: slow transit constipation

## 1 INTRODUCTION

As a medical condition featured by an extended colonic transit time (CTT) due to decreased colonic mobility, slow transit constipation (STC) can cause stomach ache, abdominal distention, defecation troubles, perianal illness and colon cancer or cerebrovascular disorders.^1^ STC aetiology is complicated while its underlying mechanisms are still not totally recognized. Numerous researches have shown that STC aetiology is associated with the intestine smooth muscles, enteric hormones, the nervous system and neurotransmitters.^2^, ^3^

Circular RNAs (circRNAs) are a special class of endogenous RNAs with covalently linked loops. Recently, an increasing number of studies revealed that circRNAs can play essential roles in tumour development.^4^ For examples, past studies of osteosarcoma have shown that the expression of circRNA was substantially changed, indicating that some circRNAs play vital roles in the development and metastasis of osteosarcoma.^5^, ^6^ Although miR‐19a is stabilized by circ_ORC2, it can activate the Akt signalling by inhibiting the expression level of PTEN, a downstream circ_ORC2 target. MiR‐19a is also up‐regulated in numerous malignant tumours to regulate the PTEN and PI3K/Akt signalling.^7^, ^8^ It was found that circ_ORC2 can promote osteosarcoma growth and invasion via the miR‐19a/Akt/PI3K/PTEN signalling.^9^

Among different miRNAs, miR‐19a is a well‐known member of the mir‐17‐92 family and was shown to function in cell proliferation, survival, differentiation and angiogenesis.^10^, ^11^ In CRC, miR‐19a was also reported to be considerably overexpressed.^12^ Furthermore, miR‐19a is induced in the presence of PRL‐3 to enhance the metastasis and proliferation of CRC cells by targeting the expression level of TG2.^13^, ^14^ Moreover, miR‐19a is related to the lymph node metastasis and EMT of CRC.^12^

As a peptide of 13 amino acids initially extracted from bovine hypothalamus, neurotensin (NT) acts as both a neurotransmitter and a neuromodulator in the central nervous system and in the periphery neurons.^15^ The pharmacological and biochemical roles of NT in peripheral and central nervous systems have actually been richly documented: as a neuromodulator controlling, the transmission of dopamine and the secretion of hormones from the anterior pituitary gland, NT exerts a powerful hypothermic and analgesic effect. In the periphery neurons, NT acts as a paracrine and endocrine modulator of the intestines.^16^, ^17^ It was suggested previously that NT can act through both the colonic smooth muscles and NANC excitatory nerves, and a reduction in the level of NT plays an essential function in colon dysmotility.^18^

As a polypeptide containing more than 20 amino acids, motilin is primarily produced by M‐cells located in the crypts of proximal terminals of nerve tissues including pineal gland, anterior pituitary, cerebral cortex, and hypothalamus.^19^ As a hormonal richly distributed in the brain and gut, motilin acts as a powerful excitatory compound when delivered iontophoretically to nerve cells in the spinal cord and cerebral cortex.^20^ The release of motilin is controlled by neuropsychic factors after eating, and it was determined that the level of motilin in STC patients after fasting was lower than that in healthy subjects, and the difference could be caused by different diet regimens.^21^, ^22^, ^23^

It was reported that neurotensin and motilin could be utilized to identify STC due to the evident changes in the circulating profiles of these GI peptides in STC patients.^24^ Moreover, circORC2 was previously reported to enhance the regulatory effect of miR‐19a.^9^ And according to the results from miRNA database investigation, miR‐19a is predicted to be correlated with the regulation of motilin expression. Therefore, in this study, we aimed to investigate the role of circORC2 in modulating miR‐19a and its downstream signalling during the pathogenesis of STC.

## 2 MATERIALS AND METHODS

### 2.1 Patient recruitment

In this study, three groups of patients were set up: (a) healthy control (HC) group (N = 48); (b) normal transit constipation (NTC) group (N = 42); and (c) slow transit constipation (STC) group (N = 34). The basic information of the patients, including their sex, age and BMI, was collected. In addition, the peripheral blood samples were collected from the patients in each group at different time points, that is 0 minute (before meal), 30 minutes, 60 minutes, 90 minutes, 120 minutes, 150 minutes and 180 minutes after meals, to assay the levels of neurotensin (NST), motilin (MLN), CRF and somatostatin. During the study, all patients were given standard meals containing 378 kcal of total energy per meal, including 57 g (61%) of carbohydrates, 14 g (33%) of fats and 6 g (6%) of proteins. The inclusion and exclusion criteria of this study were as follows. Inclusion criteria: The patients should meet the Rome III criteria used in the judgment of functional constipation^25^; each patient should have undergone at least 1 GI imaging examination, such as sigmoidoscopy, colonoscopy or abdominal ultrasound examination, throughout the past 5 years; all patients should have an age of 19 to 70 years old. Exclusion criteria were as follows: patients with organic constipation, IBS, metabolic disorders, GI disorders, enteric disorders, muscle disorders, blood disorders, a recent history of probiotics use, a recent history of major GI surgical procedures, pregnancy, a familial history of inflammatory bowel diseases or cancer, and impaired thyroid functions. Institutional ethical committee has approved the protocol of this study, and written informed consent form was acquired from all patients before the study was initiated.

### 2.2 Calculation of colonic transit time

In this study, the definitions of NTC and STC were based on the calculation of colonic transit time. In brief, each patient took 60 radiopaque markers during the study, and the radiopaque markers were randomly divided to 3 tubes, with each tube holding 20 markers. The radiopaque markers were ingested by the patients in 3 consecutive days, one tube on each day at 12:00 pm, so that routine abdominal imaging could be carried out based on a reported method^26^, ^27^ to calculate CTT. In this study, the patients having a CTT value of < 40 hours were judged as with a normal transit time (NTC),^26^ whereas the patients having a CTT value of > 68 hours were judged as with an STC.

### 2.3 Gut peptides

Peripheral blood samples at baseline were acquired from all patients after at least 12 hours of fasting. Additionally, more peripheral blood samples were acquired at 30 minutes, 60 minutes, 90 minutes, 120 minutes, 150 minutes, and 180 minutes after the patients took a standard meal as described above. All peripheral blood samples were acquired using ice‐cold blood collection tubes containing a final concentration of 1.0 mg/mL of EDTA and 500 KIU/mL of aprotinin (Sigma Aldrich). The contents of neurotensin, CRF, motilin and somatostatin in each peripheral blood sample were determined by ELISA assay kits (Thermo Fisher Scientific).

### 2.4 Cell culture and transfection

SMCs and VSMCs (ATCC, Manassas, VA) were maintained in DMEM supplemented by 10% of foetal bovine serum, 100 U/mL of penicillin, and 100 μg/mL of streptomycin (all cell culture reagents were acquired from Gibco, Thermo Fisher Scientific). The cells were maintained in a 37°C incubator under 5% CO_2_ and passaged every 3 days via digestion with 0.25% of trypsin. In this study, to study the effects of circORC2 on the expression levels of miR‐19a, NST mRNA, and MLN mRNA, SMCs and VSMCs were divided into 2 cell models, as shown below. In cell model 1, SMCs and VSMCs were divided into 2 groups, that is (a) Empty vector group (SMCs and VSMCs transfected with an empty vector) and (b) P‐circORC2 group (SMCs and VSMCs transfected with the vector carrying circORC2). In cell model 2, SMCs and VSMCs were also divided into 2 groups, that is (a) NC group (SMCs and VSMCs transfected with a negative control), and (b) CircORC2 siRNA group (SMCs and VSMCs transfected with circORC2 siRNA). In both cell models, SMCs and VSMCs were transfected with corresponding plasmids using Lipofectamine 2000 (Invitrogen) in accordance with the recommended transfection protocol shown in the manufacturer manual. At 48 hours after transfection, the cells were harvested for subsequent analyses.

### 2.5 RNA isolation and real‐time PCR

In the first step, total RNA in each sample was isolated by using a Trizol (Invitrogen) reagent in accordance with the recommended assay protocol shown in the manufacturer manual. In the next step, to detect the levels of NST mRNA and MLN mRNA in the samples, the isolated total RNA was treated with Dnase and then reversely transcribed into cDNA by using a MultiScribe RT assay kit (Thermo Fisher Scientific) in accordance with the recommended assay protocol shown in the manufacturer manual, followed by real‐time PCR carried out in a Light Cycler 480 real‐time PCR machine (Roche) in conjunction with a SYBR Green qPCR assay kit (ABI) in accordance with the recommended assay protocol shown in the manufacturer manual. Alternatively, to detect the levels of circORC2 and miR‐19a in the samples, a miRNA stem‐loop qRT‐PCR assay (Thermo Fisher Scientific) was used in accordance with the recommended assay protocol shown in the manufacturer manual. Finally, the relative expression of circORC2, miR‐19a, NST mRNA and MLN mRNA in each sample was calculated using the Ct values of real‐time PCR amplification curves following a standard method.^28^

### 2.6 Vector construction, mutagenesis and luciferase assay

To investigate the regulatory relationship between miR‐19a expression and the expression levels of MLN mRNA and NST mRNA, we first carried out a computational analysis with www.mirdb.org. As indicated by the results of the computational analysis, a putative miR‐19a binding site was identified in the 3’UTRs of MLN mRNA and NST mRNA, respectively. To further confirm the above regulatory relationship between miR‐19a expression and the expression levels of MLN mRNA and NST mRNA, we then conducted luciferase assays in SMCs and VSMCs by establishing mutant type and wild‐type vectors of MLN mRNA and NST mRNA, respectively. In brief, the 3’UTRs of MLN mRNA and NST mRNA containing the putative miR‐19a binding sites were cloned into pcDNA luciferase vectors (Promega), respectively, in accordance with the recommended cloning protocol shown in the manual of the vector manufacturer to establish the wild‐type vectors of MLN mRNA and NST mRNA. Then, a Quick Change mutagenesis assay kit (Stratagene) was used in accordance with the recommended operating protocol shown in the manufacturer manual to generate site‐directed mutagenesis mutations in the putative miR‐19a binding sites in the 3’UTRs of MLN mRNA and NST mRNA, respectively. In the next step, the mutant type 3’UTRs of MLN mRNA and NST mRNA were also cloned into pcDNA luciferase vectors, respectively, to establish the mutant type vectors of MLN mRNA and NST mRNA. Finally, SMCs and VSMCs were co‐transfected with miR‐19a mimics in conjunction with mutant type or wild‐type vectors of MLN mRNA and NST mRNA, respectively, by using Lipofectamine 2000, and the luciferase activity of transfected cells was assayed 48 hours later by using a Dual Luciferase Reporter Gene Assay kit (Promega) in accordance with the recommended assay protocol shown in the manufacturer manual. Similarly, to study the effect of circORC2 on miR‐19a expression, mutant type and wild‐type vectors of miR‐19a containing the putative binding site for circORC2 were cloned into pcDNA luciferase vectors, respectively, to establish the wild‐type and mutant type vectors of miR‐19a, which were co‐transfected with circORC2 mimics into SMCs and VSMCs using Lipofectamine 2000, and the luciferase activity of transfected cells was assayed 48 hours later by using the Dual Luciferase Reporter Gene Assay kit.

### 2.7 Western blot analysis

A RIPA lysis buffer was used to extract protein content from collected samples, and the protein was resolved by 10% SDS‐PAGE electrophoresis, transferred onto polyvinylidene difluoride (PVDF) membranes and blocked with 5% skim milk before the PVDF membranes were probed with anti‐NST and anti‐MLN primary antibodies and HRP‐labelled secondary antibodies (Abcam) in accordance with the recommended incubation conditions shown in the manual of antibody manufacturer. Then, after colour development by using an enhanced chemiluminescence (ECL) assay kit (Thermo Fisher Scientific) in accordance with the recommended assay protocol shown in the manufacturer manual, the protein band images were analysed by utilizing ImageJ software to calculate the relative protein expression of NST and MLN in each sample.

### 2.8 Statistical analysis

SPSS 21.0 for windows (SPSS) was used for all statistical analyses. A *P* value of <.05 was taken into consideration as statistically significant. All measurement data were shown as mean ± standard deviations and inter‐group comparisons were done by utilizing one‐way ANOVA along with independent‐sample *t* tests.

## 3 RESULTS

### 3.1 NST and MLN levels were most reduced in the STC group

**FIGURE 1:**
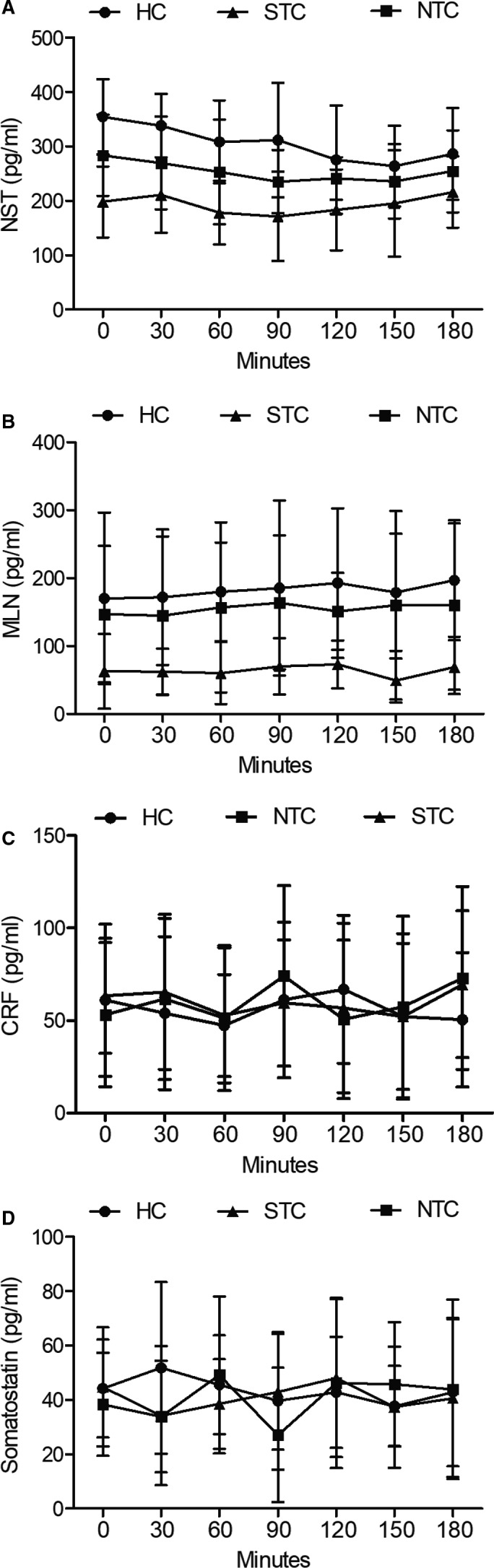
NST and MLN were most reduced in the STC group. A, NST concentrations of HC, NTC and STC groups recorded at different time points after meals; B, MLN concentrations of HC, NTC and STC groups recorded at different time points after meals; C, CRF concentrations of HC, NTC and STC groups recorded at different time points after meals; D, Somatostatin concentrations of HC, NTC and STC groups recorded at different time points after meals

Three groups of patients were set up in this study as HC group, NTC group and STC group. The basic information of these patients, including their sex, age and BMI, was listed in Table 1 and showed no significant differences among HC, NTC and STC groups. In addition, the levels of neurotensin (NST), motilin (MLN), CRF and somatostatin were also measured in the peripheral blood samples collected from each group at different time points after meals. As shown in Figure 1A, NST release was decreased in NTC patients compared with that in the HC group, whereas STC patients showed the most reduced NST level among all groups. Also, MLN concentration, as shown in Figure 1B, was markedly different among all groups, with the HC group showing the highest concentration and the STC group showing the lowest concentration. However, different from NST and MLN, the concentrations of CRF (Figure 1C) and somatostatin (Figure 1D) were similar among different groups.

**TABLE 1:** Patient basic information

Characteristics	HC (N = 48)	NTC (N = 42)	STC (N = 34)	*P* value
Age, y	73.9 ± 4.8	74.1 ± 6.5	73.1 ± 6.5	.362
Male/female	31/17	25/17	24/10	.451
BMI (kg/m^2^)	21.9 ± 3.5	22.3 ± 2.5	21.6 ± 2.3	.339

### 3.2 MiR‐19a was increased whereas circORC2 was decreased in the STC group

**FIGURE 2:**
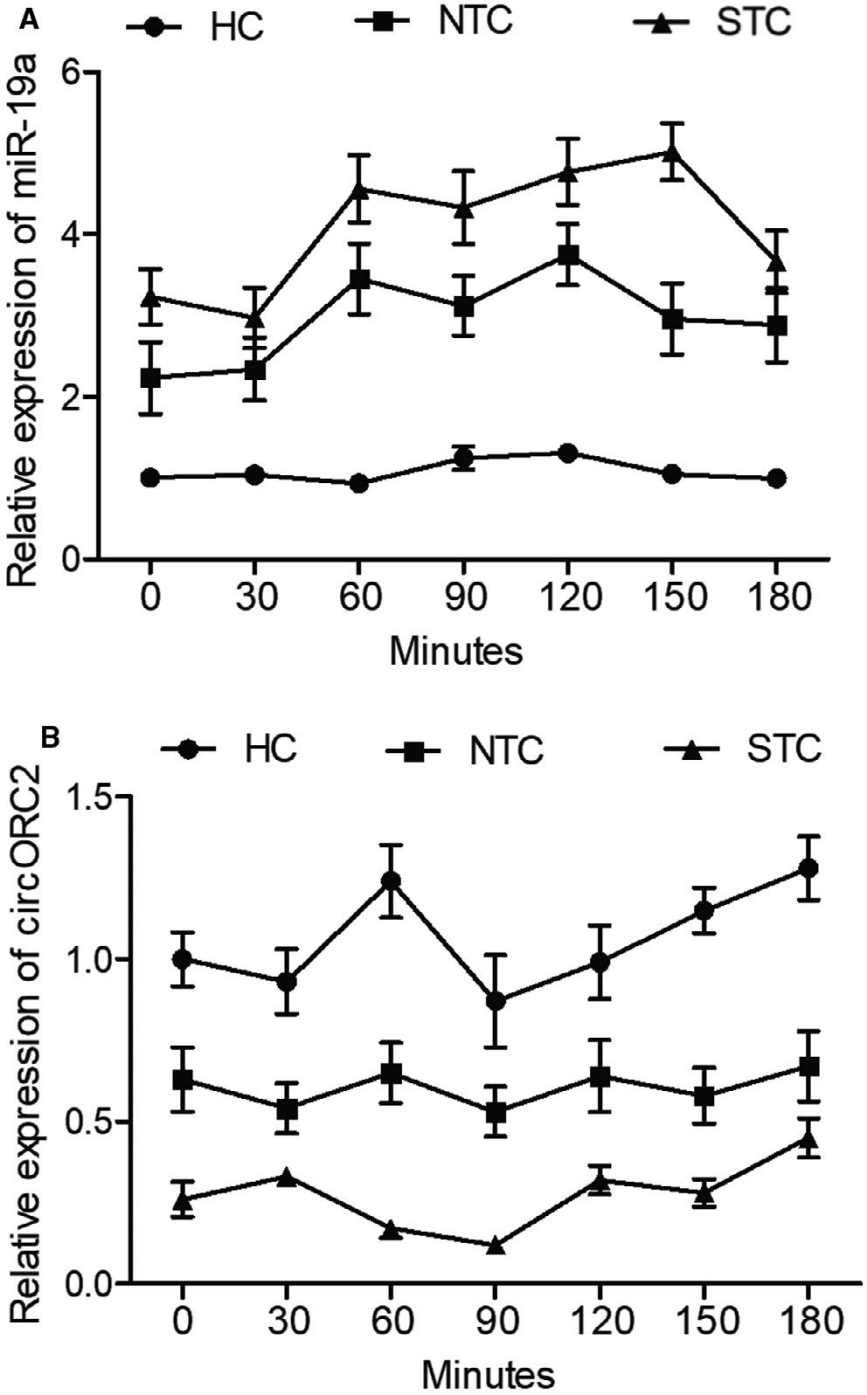
MiR‐19a was increased, whereas circORC2 was decreased in the STC group. A, Relative expression of miR‐19a in HC, NTC and STC groups recorded at different time points after meals; B, Relative expression of circORC2 in HC, NTC and STC groups recorded at different time points after meals

The expression of miR‐19a and circORC2 in the patients was observed at different time points after meals. As shown in Figure 2A, the level of miR‐19a was increased in the NTC group but was the highest in the SCT group. On the contrary, as indicated in Figure 2B, circORC2 expression was suppressed in the NTC group but was the lowest in the SCT group. Therefore, a potential negative correlation between miR‐19a and circORC2 was established.

### 3.3 MiR‐19a is targeted by circORC2

**FIGURE 3:**
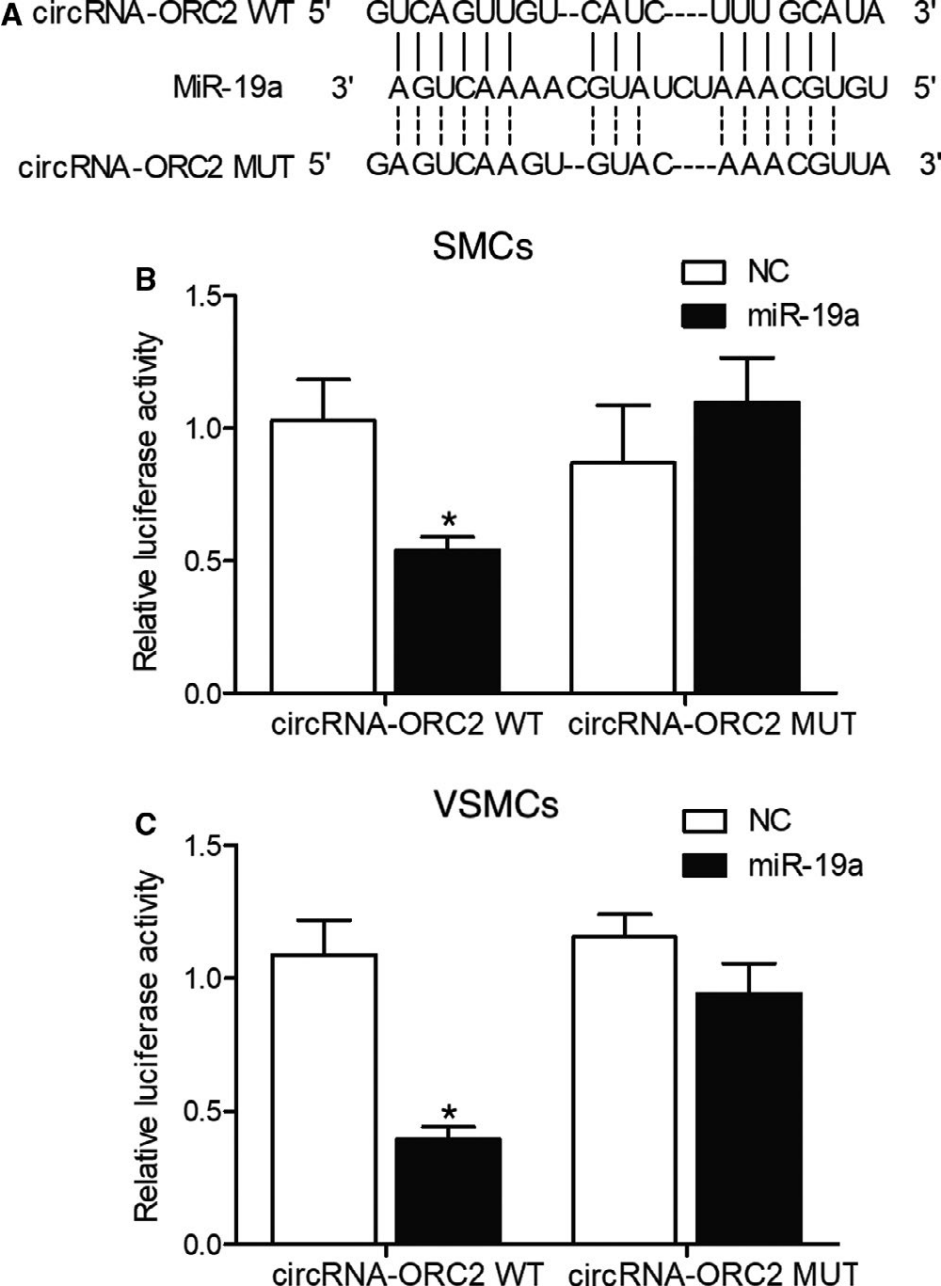
CircORC2 could target miR‐19a (^*^ *P* value <.05 compared with NC). A, Computational analysis of circORC2 and miR‐19a; B, Relative luciferase activity was reduced in SMCs co‐transfected with wild‐type circORC2 and miR‐19a; C, Relative luciferase activity was reduced in VSMCs co‐transfected with wild‐type circORC2 and miR‐19a

As shown in Figure 3A, the computational analysis of the sequences of circORC2 and miR‐19a showed a putative binding site between the two. In the subsequent luciferase assay in SMCs (Figure 3B), the luciferase activity of wild‐type circORC2 but not that of mutant circORC2 was evidently inhibited in the presence of miR‐19a. Similarly, the luciferase activity of wild‐type circORC2 in VSMCs was also reduced (Figure 3C). Therefore, it could be concluded that circORC2 could sponge miR‐19a.

### 3.4 MLN and NST mRNAs were both targeted by miR‐19a

**FIGURE 4:**
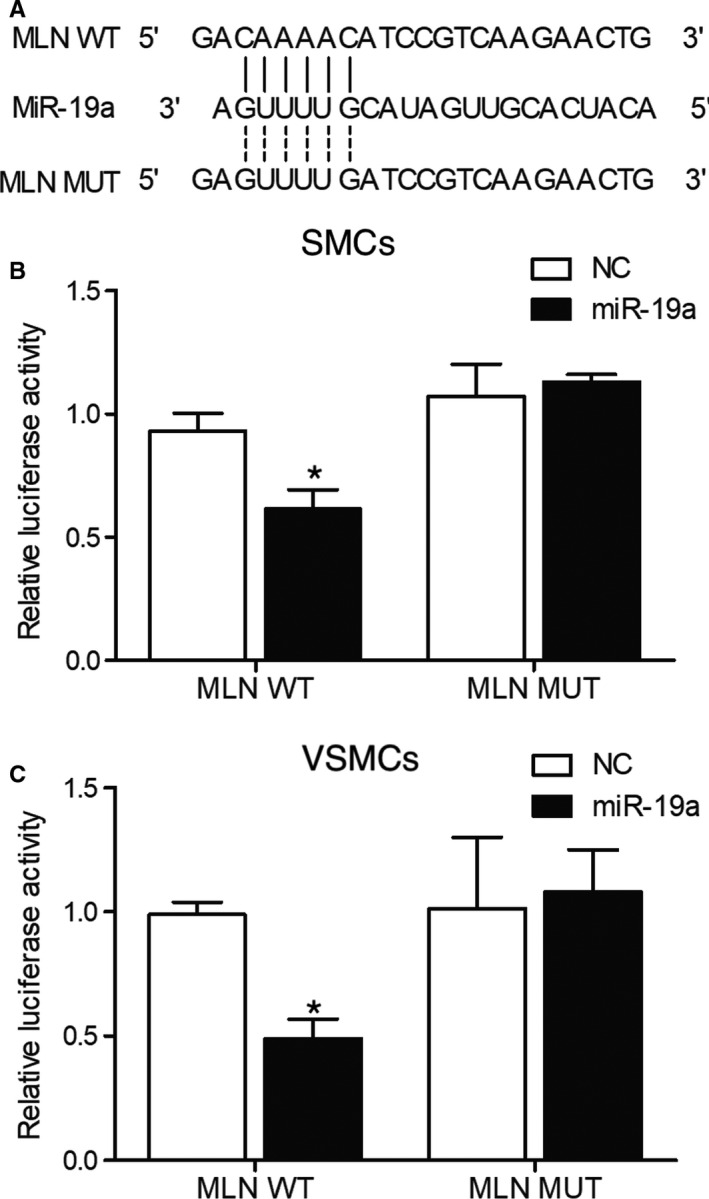
MLN mRNA was targeted by miR‐19a (^*^ *P* value <.05 compared with NC). A, Computational analysis of MLN 3’UTR and miR‐19a; B, Relative luciferase activity was reduced in SMCs co‐transfected with wild‐type MLN 3’UTR and miR‐19a; C, Relative luciferase activity was reduced in VSMCs co‐transfected with wild‐type MLN 3’UTR and miR‐19a

**FIGURE 5:**
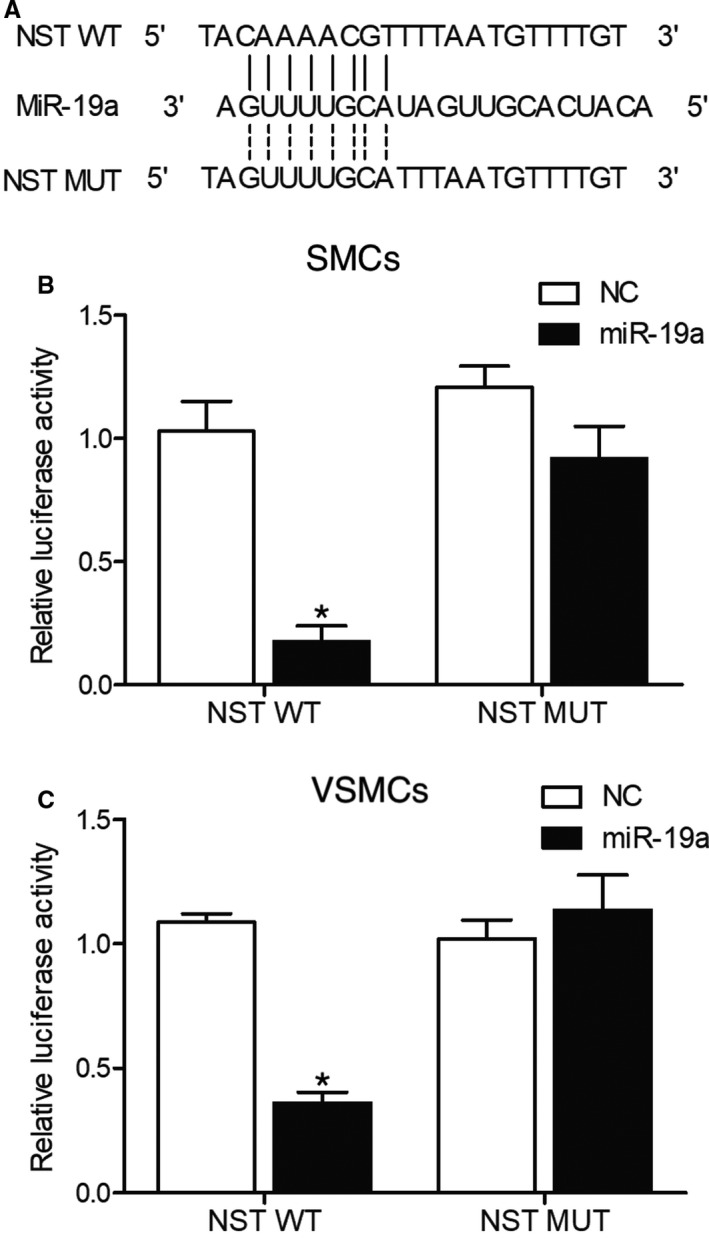
NST mRNA was targeted by miR‐19a (^*^ *P* value <.05 compared with NC). A, Computational analysis of NST 3’UTR and miR‐19a; B, Relative luciferase activity was reduced in SMCs co‐transfected with wild‐type NST 3’UTR and miR‐19a; C, Relative luciferase activity was reduced in VSMCs co‐transfected with wild‐type NST 3’UTR and miR‐19a

As indicated by the computational analysis, a putative miR‐19a binding site was identified in the 3’UTRs of MLN mRNA (Figure 4A) and NST mRNA (Figure 5A), respectively. We then conducted luciferase assays in SMCs to confirm the relationship between MLN/NST mRNA and miR‐19a. Compared with other groups, the luciferase activity in SMCs co‐transfected with miR‐19a and wild‐type MLN 3’UTR (Figure 4B) or wild‐type NST 3’UTR (Figure 5B) was the lowest. Moreover, similar results were observed in VSMCs (Figures 4C and 5C), thus indicating that MLN and NST mRNAs were both targeted by miR‐19a.

### 3.5 CircORC2 down‐regulated miR‐19a and up‐regulated MLN and NST

**FIGURE 6:**
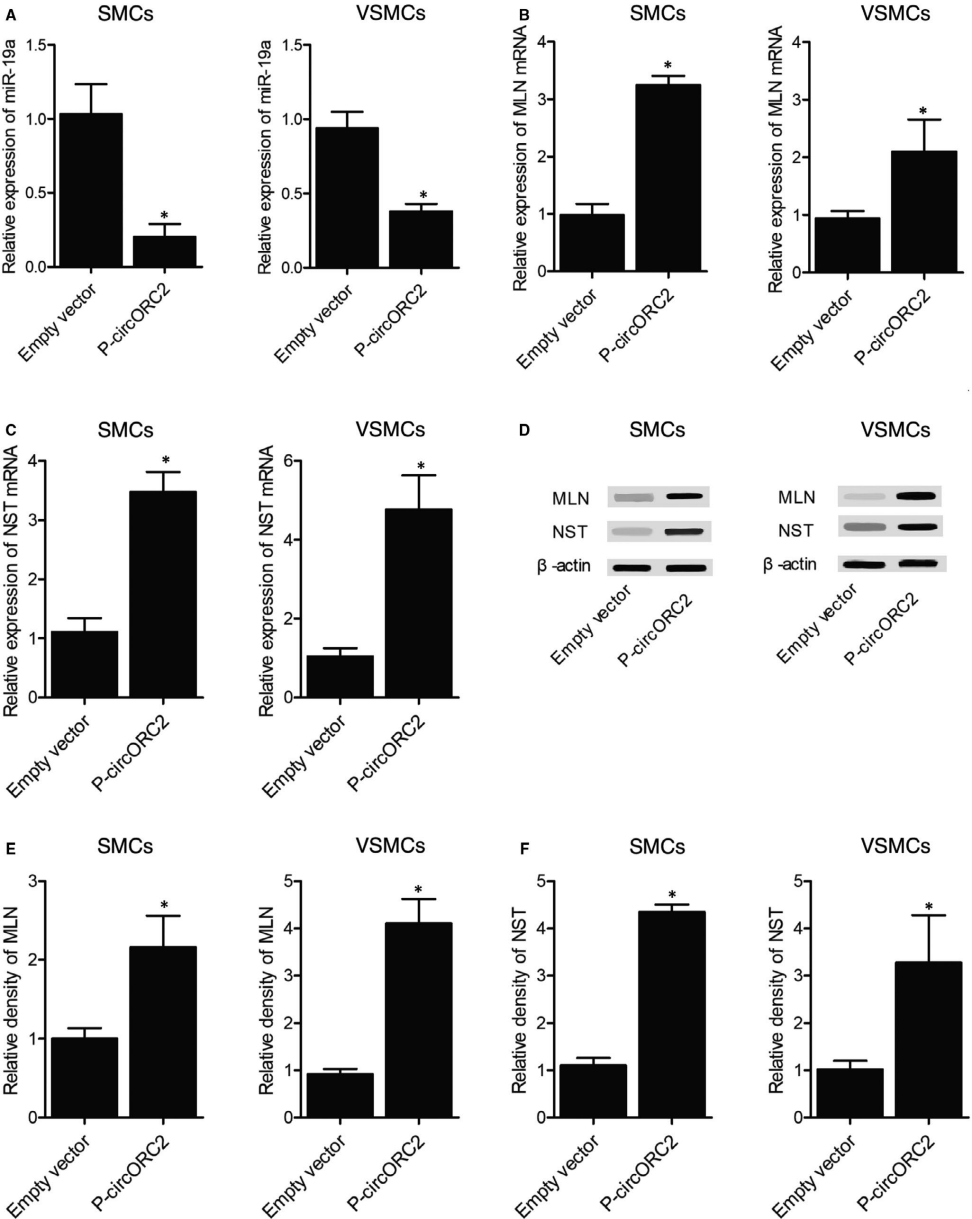
Transfection of circORC2 down‐regulated miR‐19a and up‐regulated MLN and NST (^*^ *P* value <.05 compared with Empty vector). A, Relative expression of miR‐19a was down‐regulated by the transfection of circORC2; B, Relative expression of MLN mRNA was up‐regulated by the transfection of circORC2; C, Relative expression of NST mRNA was up‐regulated by the transfection of circORC2; D, Western blotting of MLN and NST expression; E, Relative density of MLN protein was increased by the transfection of circORC2; F, Relative density of NST protein was increased by the transfection of circORC2

To further clarify the relationship between circORC2, miR‐19a, MLN and NST, the cells were transfected with empty vectors (as the control group) or vectors carrying circORC2 (as the P‐circORC2 group), respectively. As shown in Figure 6, the relative expression of miR‐19a was decreased in the P‐circORC2 group (Figure 6A), whereas MLN mRNA (Figure 6B) and NST mRNA (Figure 6C) were both significantly up‐regulated by the transfection of circORC2. Accordingly, Western blotting also showed increased MLN (Figure 6D and 6E) and NST (Figure 6D and 6F) expression in the P‐circORC2 group compared with that in the control group.

**FIGURE 7:**
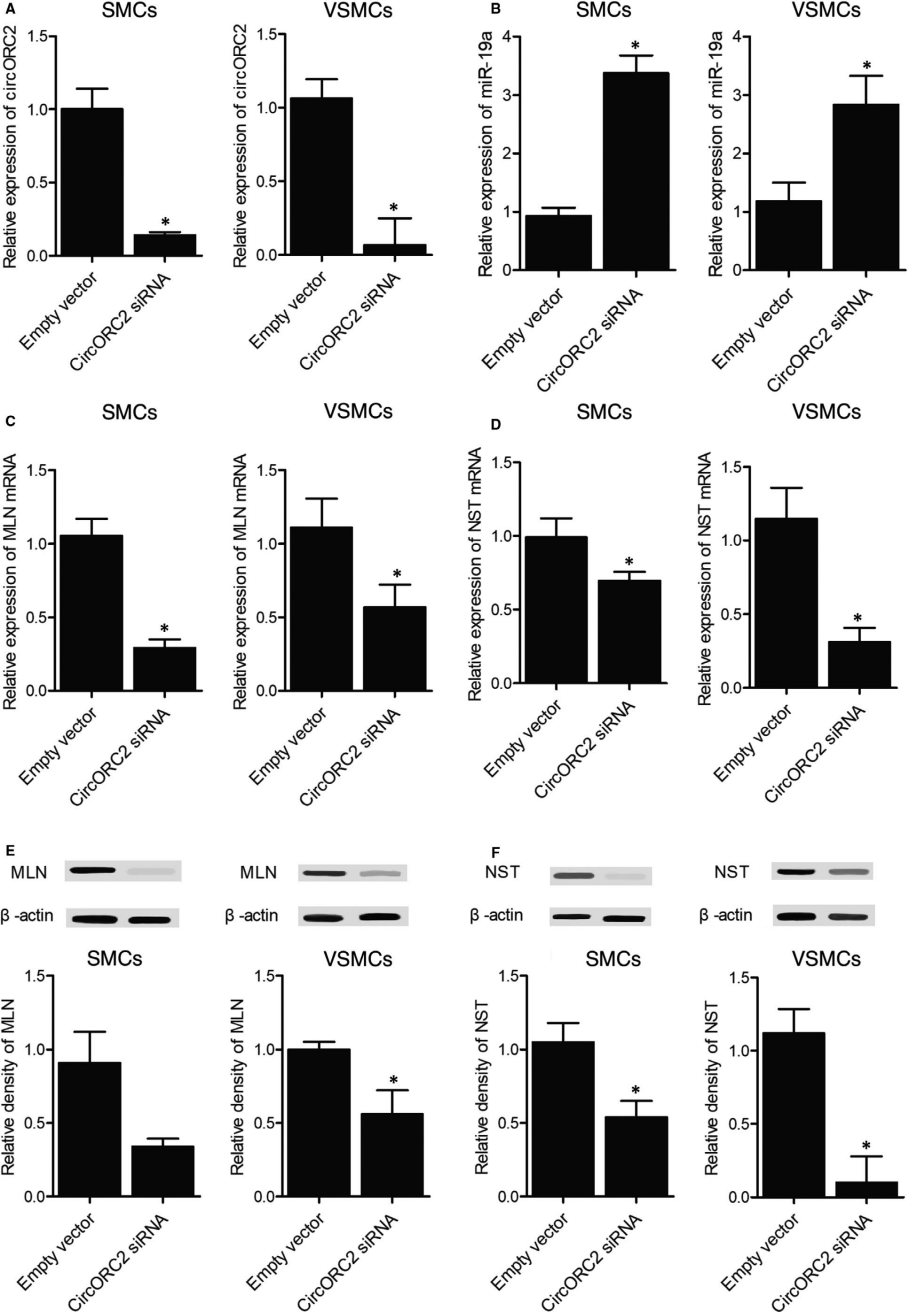
Transfection of circORC2 siRNA up‐regulated miR‐19a and down‐regulated circORC2, MLN and NST (^*^ *P* value <.05 compared with Empty vector). A, Relative expression of circORC2 was down‐regulated by the transfection of circORC2 siRNA; B, Relative expression of miR‐19a was up‐regulated by the transfection of circORC2 siRNA; C, Relative expression of MLN mRNA was down‐regulated by the transfection of circORC2 siRNA; D, Relative expression of NST mRNA was down‐regulated by the transfection of circORC2 siRNA; E, Relative density of MLN protein was reduced by the transfection of circORC2 siRNA; F, Relative density of NST protein was reduced by the transfection of circORC2 siRNA

Also, the cells were transfected with an empty vector (as the control group) or circORC2 siRNA (as the circORC2 siRNA group), respectively. As shown in Figure 7A, the relative expression of circORC2 was dramatically reduced in the presence of circORC2 siRNA, indicating the successful transfection of circORC2 siRNA. On the contrary, circORC2 siRNA transfection evidently up‐regulated the expression of miR‐19a in SMCs and VSMCs (Figure 7B). In addition, the levels of MLN mRNA (Figure 7C) and protein (Figure 7E), and the levels of NST mRNA (Figure 7D) and protein (Figure 7F), were inhibited in the circORC2 siRNA group.

Therefore, the signalling pathway of circORC2, miR‐19a and neurotensin/motilin was established, which indicated that the overexpression of circORC2 could up‐regulate the levels of neurotensin and motilin, thus exerting a beneficial effect during the treatment of STC.

## 4 DISCUSSION

STC is characterized by an elevated value of CTT as determined by radio‐markers using radio‐nucleotide techniques. In fact, reduced colonic motility has become an essential pathophysiological factor in the prognosis of STC. Lately, numerous researches have shown that the microbiota in the gut and intestines may be involved in the pathogenesis of constipation disorders.^29^, ^30^ In this study, we enrolled three groups of patients as HC group, NTC group and STC group. We found that the levels of NST and MLN were both markedly decreased in NTC and STC groups, especially in the STC group. Also, miR‐19a was most increased, whereas the level of circORC2 was the lowest in the STC group.

Lately, it was discovered that circ_ORC2 is overexpressed in the cells of osteosarcoma to play an essential and regulatory role by acting as a sponge of microRNAs.^31^, ^32^ Furthermore, it was shown that circ_ORC2 contains a miR‐19a binding site.^33^ In this study, circORC2 was validated to sponge the expression of miR‐19a by the computational analysis and luciferase assay. MLN and NST mRNAs were both targeted by miR‐19a. In addition, we found that the transfection of circORC2 down‐regulated miR‐19a and up‐regulated the expression of MLN and NST in SMCs and VSMCs. On the contrary, the transfection of circORC2 siRNA dramatically reduced the relative expression of circORC2 but evidently up‐regulated the expression of miR‐19a in SMCs and VSMCs, whereas the levels of MLN and NST were reduced in the circORC2 siRNA group.

Motilin has been shown to boost the rate to empty the stomach after a mean in both humans and rats.^34^, ^35^ In fact, motilin can be of a significant value in regulating gastrointestinal mobility because it is able to empty the stomach for the following meal.^36^ Nonetheless, constipation is a complicated disease that can involve various factors. For example, hormonal levels in the body can affect the physiological behaviour of constipation. Thus, the presence of inhibitory neurotransmitters such as NO, AVP and NOS or the lack of excitatory neurotransmitters such as SP may hinder colonic mobility, hence resulting in a slowed CTT.^37^, ^38^ A number of receptors of inhibitory and excitatory neurotransmitters are present in the colon to promote bowel movement.^38^, ^39^

It was likewise found that circ_ORC2 enhances the invasion and growth of osteosarcoma cells via the miR‐19a/PI3K/Akt/PTEN signalling, suggesting that circ_ORC2 can interact with miR‐19a to inhibit PTEN expression while activating the Akt signalling.^9^ In this study, we established the circORC2/miR‐19a/neurotensin/motilin signalling pathway, which indicated that the overexpression of circORC2 up‐regulated the level of neurotensin and motilin, thus exerting a beneficial effect during the treatment of STC.

Neurotensin (NT) was initially derived from bovine hypothalamus and showed a vasodilation effect in rats.^15^ Subsequent studies have identified the role of NT and neuromedin (NMN) in both the central and peripheral nervous systems.^40^ Past researches revealed the broad involvement of NT in the nerve systems through neurotensin and dopamine receptors to play a role in numerous brain conditions including Huntington's diseases and schizophrenia.^41^, ^42^ The research on the neuroendocrine status in STC patients revealed that several peptides, such as motilin, neurotensin, and somatostatin, may change colonic mobility.^43^, ^44^, ^45^ By evaluating distal and proximal GI hormones in STC patients under fasting and postprandially, it was shown that the levels of proximal GI hormones such as cholecystokinin and gastrin were elevated along with reduced levels of distal GI hormones such as neurotensin and polypeptide YY.^44^ In addition, SR 48 692, a neurotensin antagonist, promoted the emptying of the stomach and defecation.^46^

However, there are limitations of our study. The patient number recruited is limited. And the limited sample size will influence the outcome and accuracy of the study. Also, the establishment of signalling pathway should be further validated in vivo. Therefore, in our future study, larger sample size with varied populations, and validation of the signalling pathway in vivo, is necessary.

## 5 CONCLUSION

Our study established the circORC2/miR‐19a/neurotensin/motilin signalling pathway, which indicated that the overexpression of circORC2 up‐regulated the level of neurotensin and motilin, thus exerting a beneficial effect during the treatment of STC.

## AUTHOR CONTRIBUTION

**Yuan‐yuan Wang:** Conceptualization (lead); Data curation (equal); Methodology (equal); Software (equal); Validation (equal); Writing‐original draft (equal). **Ruiyun Lu:** Data curation (equal); Investigation (equal); Methodology (equal); Resources (equal); Software (equal); Visualization (equal); Writing‐original draft (equal). **Ji Shi:** Investigation (equal); Software (equal); Validation (equal); Visualization (equal). **Shuai Zhao:** Investigation (equal); Methodology (equal); Resources (equal); Software (equal). **Xia Jiang:** Investigation (equal); Resources (equal); Software (equal); Validation (equal); Writing‐original draft (equal). **Xiaosong Gu:** Formal analysis (equal); Methodology (equal); Project administration (equal); Supervision (equal); Writing‐original draft (equal); Writing‐review & editing (equal).

## Data Availability

The data that support the findings of this study are available from the corresponding author upon reasonable request.
